# Executive functions in university students from Chile and Ecuador: Dataset

**DOI:** 10.1016/j.dib.2025.112127

**Published:** 2025-10-10

**Authors:** Carlos Ramos-Galarza, Diego D. Díaz-Guerra, Marena de la C. Hernández-Lugo, Mónica Bolaños-Pasquel, Yunier Broche-Pérez

**Affiliations:** a Centro de Investigación MIST, Facultad de Psicología. Universidad Indoamérica. Quito, Ecuador; b Psychology Department, Universidad Central Marta Abreu de Las Villas. Villa Clara, Cuba; c Prisma Behavioral Center, FL, USA

**Keywords:** Executive functions, UEF-1 scale, University students, Monitoring, Latin america

## Abstract

Executive functions are high-level cognitive abilities that enable individuals to regulate their behavior and thought processes consciously. In the university setting, students must effectively utilize these functions to achieve success in both their personal and academic development. The key executive functions relevant to the university context include: the supervisory attentional system, deliberate emotional regulation, conscious monitoring of responsibilities, behavior verification for learning, task organization, conscious behavior regulation, and decision-making. This dataset comprises 1373 surveys from university students in Ecuador and Chile, aged between 17 and 33 years (M_age_ = 20.53, SD = 2.34). It includes descriptive statistical values for the sample, item responses, and detailed descriptions of each evaluated variable. This database is designed to facilitate further analysis of the Executive Functions Scale and the role of these cognitive skills in the university environment.

Specifications Table

**Table:** 

Subject	Neuropsychology and Physiological Psychology, education.
Specific subject area	*This database was obtained in the context of university education and describes the executive functions of students at this educational level.*
Type of data	Table.
Data collection	The study started with authorization from the Research Ethics Committee of the Pontificia Universidad Católica del Ecuador. Informed consent was subsequently obtained, along with the application of the Executive Functions Scale in the university context, between June 2022 and January 2023. The instrument was administered in three cities: Antofagasta and Maule in Chile, and Quito in Ecuador. Data collection took place in the students' classrooms. Participation was voluntary, and students could withdraw from the study at any time.
Data source location	(a) Pontificia Universidad Católica del Ecuador, Quito, Ecuador (−0.20922188068183684, −78.49094017773747) (b) Universidad Indoamérica, Quito, Ecuador (−0.120836066014451, −78.49834373116846) (c) Universidad de Antofagasta, Antofagasta, Chile (−23.528090679848287, −70.2540504176277) (d) Universidad Católica del Maule, Maule, Chile (−35.42241125007197, −71.61716918234872)
Data accessibility	Repository name: Mendeley Data Data identification number: DOI: 10.17632/gfrmzgmkbt.2 Direct URL to data: https://data.mendeley.com/datasets/gfrmzgmkbt/2 Instructions for accessing these data: Open access database.
Related research article	*The database presented is related to the article:* Ramos-Galarza, C., Ramos, V., Del Valle, M., Lepe-Martínez, N., Cruz-Cárdenas, J., Acosta-Rodas, P., Bolaños-Pasquel, M. Executive functions scale for university students: UEF-1. Frontiers in Psychology, 14: 1192,555, (2023). https://doi.org/10.3389/fpsyg.2023.1192555 [1]*.*

## 1 Value of the Data

• This dataset captures executive functioning profiles of university students from Chile and Ecuador, offering a cross-national perspective on cognitive and self-regulatory processes in higher education.
• It provides detailed insights into core executive functions, including supervisory attentional control, deliberate emotional regulation, conscious monitoring of responsibilities, behavioral verification for learning, task management, conscious behavioral regulation, and decision-making.
• The dataset is particularly valuable for instrument developers seeking to validate or refine assessment tools for executive functioning in Latin American university contexts.
• It is also useful for cross-national comparativists interested in testing measurement invariance and comparing executive function constructs across cultural settings.
• Policy analysts and educational decision-makers may use the data to inform targeted interventions and support strategies for cognitive development in university populations.
• The dataset can contribute to the establishment of normative data, the development of new executive function assessment scales, and hypothesis-driven research in psychology and education.

## 2 Background

This study is theoretically framed within Barkley's model of Self-Regulation [2], which offers a comprehensive perspective of executive functions (EFs) by reconceptualizing them as goal-directed behaviors mediated by self-control. According to this model, EFs are not merely ``cold'' cognitive skills, but constitute a self-regulatory system that enables the management of behavior to achieve future goals. This approach is particularly suitable for the university context, where academic success critically depends on the student's ability to organize, direct, and monitor their behavior in the absence of constant external supervision [3]. Skills such as monitoring responsibilities, attentional control, and behavior regulation are fundamental to academic success [4,5]. By facilitating effective planning, organization, and self-evaluation, these cognitive abilities enhance learning efficiency and, consequently, academic performance [6].

According to this framework, Barkley identifies key components that translate into measurable behavioral dimensions. Consequently, for the operationalization in this study, the model's constructs guided the definition of seven specific dimensions. Thus, the dimension of *Conscious regulation of behavior* is central to the model, representing the inhibition of inadequate responses and the initiation of goal-oriented actions. In a complementary manner, dimensions such as *resource management and Conscious monitoring of responsibilities are directly derived from the components of time management and organization inherent in* self-regulation. Similarly, *Conscious regulation of emotions* and *Decision-making* are viewed as essential applications of self-control in the emotional and problem-solving realms, respectively. For its part, the *Supervisory Attention System and Verification of Learning-Related Behavior* operationalize the component of self-monitoring and feedback on one's performance.

Therefore, this theoretical model directly guided the translation of abstract constructs into concrete self-report items. Each item on the UEF-1 scale was designed to assess the frequency or effectiveness with which students employ these self-regulatory behaviors in their daily academic lives. This transnational study, involving university students from Ecuador and Chile, aims to explore the interrelationships among these executive functions. By examining these relationships, the study seeks to provide a comprehensive understanding of how these functions impact students' holistic development across various educational environments. Insights gained from this research may offer opportunities to enhance educational practices and support mechanisms tailored to diverse academic contexts.

## 3 Data Description

The dataset comprises responses from 1373 university students from Chile and Ecuador, collected using the UEF-1 executive functions scale, which was designed specifically for university students [1]. The data collection period spanned from June 2022 to January 2023. This dataset includes contributions from three Latin American universities: Pontificia Universidad Católica del Ecuador, Universidad Católica del Maule in Chile, and Universidad de Antofagasta in Chile.

Guided by Barkley's model of self-regulation [2], the seven measured constructs were explicitly defined and operationalised for the university context. Each construct was translated into self-report items designed to capture the frequency or perceived efficacy of specific self-regulatory behaviors in academic and personal life. Below is the definition and operationalisation for each dimension:

*Conscious Regulation of Behavior (Items 3, 11, 16, 17, 18, 19):* This construct refers to the deliberate inhibition of inappropriate responses and the initiation of actions aligned with long-term goals. Rooted in Barkley's core concept of behavioral inhibition, it was operationalised through items that measure the ability to control impulses, adhere to rules, and initiate tasks despite distractions.

*Conscious Monitoring of Responsibilities (Items 2, 8, 9, 15, 27):* Defined as the active tracking and management of obligations to ensure their fulfillment. This dimension operationalises Barkley's component of self-organization and time management. Items were designed to assess behaviors such as reviewing pending tasks, planning steps to meet deadlines, and maintaining an overview of academic responsibilities.

*Supervisory Attentional System (Items 10, 13, 14, 22, 28):* This construct refers to the cognitive mechanism that regulates focus, alertness, and the allocation of attentional resources. It translates Barkley's concept of self-directed attention into items that measure the ability to concentrate on tedious tasks, resist internal and external distractions, and efficiently shift focus between different activities.

*Verification of Learning-Related Behavior (Items 20, 23, 24, 30):* Defined as the assessment and adjustment of one's actions to optimize knowledge acquisition and academic performance. This dimension operationalises the self-monitoring and feedback function of Barkley's model. Items probe behaviors such as checking work for errors, evaluating study methods for effectiveness, and adjusting strategies based on outcomes.

*Decision-Making (Items 5, 12, 21):* This construct represents the process of evaluating alternatives and selecting a course of action based on reasoning and long-term objectives. It captures the application of self-regulation in problem-solving scenarios. Items were created to measure the tendency to analyze options, consider consequences, and avoid impulsive choices.

*Conscious Regulation of Emotions (Items 4, 25, 29, 31):* Defined as the intentional management of affective responses to maintain psychological balance and achieve goals. This dimension is grounded in Barkley's emphasis on regulating emotions as a key aspect of self-control. Items assess the ability to manage frustration, control emotional reactions in stressful situations, and maintain composure.

*Management of Resources for Task Completion (Items 1, 6, 7, 26):* This construct refers to the efficient allocation of time, effort, and materials to achieve objectives. It directly operationalizes Barkley's components of planning and organization. Items measure behaviors related to structuring study time, organizing physical and digital materials, and prioritizing tasks to ensure successful task completion.

The scale was administered in printed form in environments free from distractions to ensure accurate responses (Annex 1 presents the complete version of the scale). Participation was voluntary, and students could withdraw from the study at any time. After collecting the completed questionnaires, the data were entered into SPSS statistical software for analysis. The analyses performed included: sociodemographic indicators of participants (see Table 1), responses to each item on the scale (see Table 2), central tendency and dispersion values for each executive function (see Table 3), correlations between items on the instrument (see Table 4), and correlations among the executive functions themselves (see Table 5, Annex 1).

**Table 1:** Descriptive data of the student sample (*N* = 1373).

Country	Frequency	Percentage	Age	Gender	Educational Level
**Chile**	663	48.3 %	*M* = 20.26, *SD* = 2.43	308 women (46.46 %), 355 men (53.54 %)	*M* = 2.82, *SD* = 2.47
**Ecuador**	710	51.7 %	*M* = 20.76, *SD* = 2.19	465 women (65.49 %), 245 men (34.51 %)	*M* = 4.68, *SD* = 2.52

**Table 2:** Descriptive statistics for each item of the executive functions scale (UEF-1).

Item	Min	Max	Mean	SE	SD	Variance
1	2,00	5,00	4,35	,02	,84	,70
2	2,00	5,00	4,40	,02	,74	,55
3	1,00	5,00	4,01	,02	,82	,68
4	1,00	5,00	3,69	,02	,90	,81
5	2,00	5,00	4,29	,02	,80	,63
6	1,00	5,00	3,88	,03	,98	,97
7	1,00	5,00	4,06	,03	,95	,91
8	1,00	5,00	4,17	,02	,79	,63
9	1,00	5,00	4,26	,02	,71	,50
10	1,00	5,00	3,56	,03	,93	,86
11	1,00	5,00	3,59	,03	1,14	1,29
12	1,00	5,00	4,08	,02	,82	,67
13	1,00	5,00	3,60	,02	,91	,83
14	1,00	5,00	3,96	,02	,86	,75
15	3,00	5,00	4,55	,02	,64	,41
16	1,00	5,00	4,49	,02	,77	,60
17	1,00	5,00	4,10	,02	,86	,74
18	2,00	5,00	4,22	,02	,83	,70
19	1,00	5,00	3,94	,02	,85	,72
20	1,00	5,00	4,20	,02	,85	,73
21	1,00	5,00	3,91	,02	,85	,72
22	1,00	5,00	3,82	,02	,84	,71
23	1,00	5,00	4,24	,02	,92	,85
24	1,00	5,00	4,29	,02	,82	,68
25	1,00	5,00	3,81	,03	,99	,98
26	1,00	5,00	4,26	,03	,93	,87
27	1,00	5,00	4,21	,02	,83	,69
28	1,00	5,00	3,51	,03	1,00	1,01
29	1,00	5,00	3,67	,03	1,07	1,15
30	1,00	5,00	4,05	,02	,86	,73
31	1,00	5,00	3,75	,03	,98	,96

**Table 3:** Descriptive statistics of the executive function variables assessed in students (*N* = 1373).

Variables	Minimum	Maximum	Mean	Standard Error	Standard Deviation	Variance
Conscious Monitoring of Responsibilities	11.00	25.00	21.58	0.070	2.60	6.80
Supervisory Attentional System	6.00	25.00	18.44	0.093	3.47	12.05
Conscious Regulation of Behavior	11.00	25.00	20.42	0.076	2.84	8.07
Conscious Verification of Behavior for Learning	6.00	20.00	16.77	0.067	2.49	6.21
Decision Making	5.00	15.00	12.28	0.052	1.94	3.78
Emotion Regulation	4.00	20.00	14.92	0.085	3.17	10.07
Management of Elements to Solve Tasks	7.00	20.00	16.55	0.076	2.81	7.95

**Table 4:** Correlations among items of the executive functions scale (UEF-1).

	1	2	3	4	5	6	7	8	9	10	11	12	13	14	15	16	17	18	19	20	21	22	23	24	25	26	27	28	29	30	31
1	1																														
2	,26	1																													
3	,22	,19	1																												
4	,14	,15	,35	1																											
5	,17	,22	,17	,30	1																										
6	,47	,17	,21	,22	,23	1																									
7	,33	,19	,19	,15	,22	,56	1																								
8	,11	,36	,16	,14	,24	,16	,20	1																							
9	,20	,44	,24	,18	,24	,20	,18	,51	1																						
10	,22	,33	,24	,27	,20	,24	,17	,29	,36	1																					
11	,20	,14	,32	,26	,15	,23	,21	,17	,19	,36	1																				
12	,22	,28	,24	,33	,39	,22	,17	,22	,31	,29	,24	1																			
13	,24	,28	,24	,25	,25	,30	,21	,23	,29	,43	,27	,31	1																		
14	,28	,34	,25	,23	,26	,29	,24	,26	,35	,53	,39	,26	,44	1																	
15	,23	,31	,17	,09	,19	,20	,19	,31	,35	,22	,16	,23	,21	,22	1																
16	,22	,17	,23	,13	,20	,20	,14	,16	,24	,15	,23	,27	,16	,23	,27	1															
17	,21	,21	,22	,16	,23	,19	,16	,23	,23	,25	,28	,23	,29	,34	,23	,33	1														
18	,14	,15	,19	,18	,19	,18	,18	,18	,14	,20	,23	,20	,24	,22	,19	,22	,34	1													
19	,14	,15	,44	,27	,23	,16	,14	,16	,19	,19	,25	,26	,21	,22	,17	,21	,20	,23	1												
20	,27	,28	,23	,12	,18	,24	,21	,22	,24	,24	,19	,25	,30	,28	,26	,24	,22	,23	,26	1											
21	,18	,28	,21	,29	,47	,21	,18	,26	,30	,32	,18	,45	,32	,29	,20	,19	,23	,20	,30	,21	1										
22	,26	,35	,25	,22	,28	,31	,23	,26	,34	,55	,32	,32	,50	,53	,27	,21	,32	,25	,21	,28	,40	1									
23	,21	,18	,20	,06	,11	,19	,19	,18	,19	,18	,17	,16	,16	,21	,22	,20	,16	,22	,16	,47	,16	,22	1								
24	,23	,26	,23	,13	,16	,24	,22	,22	,29	,26	,21	,24	,28	,28	,30	,27	,26	,22	,20	,33	,21	,34	,28	1							
25	,14	,15	,31	,44	,25	,15	,15	,12	,12	,18	,30	,27	,21	,24	,12	,16	,23	,26	,33	,10	,25	,23	,08	,19	1						
26	,43	,17	,22	,13	,18	,42	,40	,13	,14	,13	,21	,17	,18	,23	,19	,18	,21	,23	,19	,22	,17	,21	,19	,25	,18	1					
27	,27	,38	,26	,14	,23	,24	,25	,29	,39	,25	,21	,25	,30	,29	,30	,19	,23	,18	,18	,30	,25	,30	,26	,34	,15	,28	1				
28	,24	,32	,22	,22	,21	,35	,27	,26	,32	,50	,32	,30	,43	,39	,22	,19	,26	,18	,19	,31	,30	,48	,22	,34	,20	,21	,37	1			
29	,17	,23	,27	,51	,33	,21	,15	,15	,25	,29	,26	,40	,27	,27	,14	,20	,18	,17	,24	,19	,34	,32	,12	,22	,46	,16	,25	,37	1		
30	,19	,31	,24	,23	,24	,25	,19	,28	,31	,26	,22	,28	,31	,29	,25	,25	,31	,24	,25	,40	,29	,36	,33	,34	,16	,22	,36	,39	,30	1	
31	,14	,16	,29	,62	,32	,19	,14	,16	,16	,22	,28	,36	,27	,26	,08	,19	,22	,25	,33	,11	,37	,23	,08	,14	,54	,16	,14	,22	,61	,25	1

**Table 5:** Correlations among executive functions.

	UEF1	UEF2	UEF3	UEF4	UEF5	UEF6	UEF7
UEF1	1						
UEF2	,543	1					
UEF3	,432	,530	1				
UEF4	,517	,499	,478	1			
UEF5	,447	,476	,408	,364	1		
UEF6	,278	,406	,455	,266	,501	1	
UEF7	,371	,416	,405	,397	,323	,265	1

### 3.1 Reliability and validity evidence of the executive functions scale (UEF-1)

The Executive Functions Scale for the university context presented satisfactory psychometric properties across its seven subscales. Specifically, the supervisory attentional system showed α = 0.85 and Ω = 0.82; conscious regulation of emotions α = 0.82 and Ω = 0.75; conscious monitoring of responsibilities α = 0.80 and Ω = 0.75; verification of behavior to learn α = 0.71 and Ω = 0.65; management of elements to solve tasks α = 0.76 and Ω = 0.72; decision making α = 0.71 and Ω = 0.65; and conscious regulation of behavior α = 0.76 and Ω = 0.74. These results indicate adequate internal consistency and reliability for each domain of executive functioning. Furthermore, the confirmatory factor analysis of the seven-factor structure revealed an acceptable fit, χ²(413) = 1649.14, *p* < 0.001, CFI = 0.91, SRMR = 0.04, and RMSEA = 0.04, supporting the factorial validity of the instrument for assessing executive functions in the university context.

## 4 Experimental Design, Materials, and Methods

### 4.1 Study design

The research design aims to analyze executive function performance within the university context. The dataset evaluates seven key executive function variables: UEF1 (Conscious Monitoring of Responsibilities), UEF2 (Supervisory Attentional System), UEF3 (Conscious Behavior Regulation), UEF4 (Behavior Verification for Learning), UEF5 (Decision-Making), UEF6 (Conscious Emotional Regulation), and UEF7 (Task Management). Previous studies have reported that executive functions significantly influence the success of university students in their professional training and their ability to regulate behavior for effective learning [7,8].

### 4.2 Materials and methods

The methodology employed in constructing this dataset is quantitative, non-experimental, and cross-sectional in nature. The study commenced following approval from the Research Ethics Committee of the Pontificia Universidad Católica del Ecuador. The initial phase involved developing the scale by drafting items designed to assess executive functions in the university context. Cognitive interviews were subsequently conducted to ensure participants understood each item. Once the instrument was refined to its final version, it was administered on a large scale.

A minimum sample size of 10 participants per item was calculated, resulting in a minimum requirement of 310 participants. However, the final sample of 1373 university students provides the statistical power needed for future studies involving structural equation modeling or psychometric analysis of the scale. The participants' ages ranged from 17 to 33 years, covering the typical age range for university studies. After analyzing participant responses, 3 % of the questionnaires were excluded due to incomplete answers or the absence of signed informed consent forms.

Data were collected in three Latin American cities: Maule and Antofagasta in Chile, and Quito in Ecuador. Both countries feature capitalist economic systems and a mix of private and public higher education institutions. These characteristics ensure that the dataset can be used to understand executive functioning among university students in similar socio-cultural contexts.

## Limitations

This study presents several limitations that should be considered when interpreting the results. One significant limitation is sample representativeness. Although the sample includes participants from three universities in Chile and Ecuador, it may not fully capture the diversity of university students across Latin America or other regions. Consequently, the findings might be influenced by the specific educational and socio-economic contexts of the participating institutions.

Another limitation is the reliance on self-reported data from the UEF-1 scale. Self-report measures can introduce biases, such as social desirability or inaccuracies in self-assessment, which may affect the validity of the findings. Furthermore, the study's context-specific nature—conducted in particular socio-cultural and educational environments in Latin America—limits the generalizability of the findings. Variations in educational systems, socio-economic conditions, and cultural norms could influence executive function performance and perceptions differently in other settings.

Environmental factors, such as peer influence or the presence of instructors, could affect participants' responses and the overall quality of the data. Addressing these limitations in future research could provide a more nuanced understanding of executive functions across diverse educational contexts, thereby improving the validity and reliability of the findings.

## Ethics Statement

Institutional review boards (IRBs) or ethics committees are responsible for ensuring that research involving human participants complies with ethical standards. Approval by such a committee confirms that the study design minimizes risks to participants and provides informed consent and data confidentiality. In this case, the Ethics Committee reviewed and approved the research protocol, assigning it the identifier 2019–58EO, which serves as an official record of ethical compliance and oversight throughout the study’s execution.

The UEF-1 scale was administered in a manner that minimized potential discomfort or distress to participants. The study was conducted in classroom settings designed to be free from distractions, ensuring accurate and thoughtful responses. The research team is committed to upholding the highest ethical standards in all phases of the study and ensuring that the rights and well-being of participants are protected throughout the research process.

## Data Accessibility and License

The dataset accompanying this article is made openly available under the terms of the Creative Commons Attribution 4.0 International License (CC BY 4.0). This license permits unrestricted use, distribution, and reproduction in any medium, provided the original author and source are properly credited.

## CRediT Author Statement

**Carlos Ramos-Galarza:** Conceptualization, Data Curation, Methodology, Supervision, Writing – Review & Editing, Project Administration. **Diego D. Díaz-Guerra:** Conceptualization, Data Curation, Validation, Writing – Review & Editing. **Marena de la C. Hernández-Lugo:** Conceptualization, Data Curation, Validation, Writing – Review & Editing. **Mónica Bolaños-Pasquel:** Conceptualization, Data Curation, Validation, Writing – Review & Editing. **Yunier Broche-Pérez:** Conceptualization, Methodology, Supervision, Writing – Review & Editing, Writing – Original Draft Preparation.

## Data Availability

Mendeley DataExecutive Functions in University Students from Chile and Ecuador: Dataset (Original data).
